# Successful treatment of hard flaccid syndrome with multimodal therapy: a case report study

**DOI:** 10.1038/s41443-024-00955-x

**Published:** 2024-07-25

**Authors:** R. Omer Yazar, Muhammed A. M. Hammad, David W. Barham, Babak Azad, Faysal A. Yafi

**Affiliations:** 1 Bagcilar Research and Training Hospital, Istanbul, Turkey; 2 Department of Urology, University of California, Irvine, CA USA

**Keywords:** Sexual dysfunction, Quality of life, Regenerative medicine

## Abstract

Our article outlines a case study assessing the use of low-intensity shock wave therapy (LiSWT) for managing Hard Flaccid Syndrome (HFS). Given the absence of standardized treatments for HFS, LiSWT could serve as an additional tool in the treatment arsenal. The case involved a 36-year-old male presenting HFS, low libido, chronic pain, and erectile dysfunction. Treatment comprised phosphodiesterase type 5 inhibitor (PDE5-I), physical therapy, and LiSWT. Following six sessions, the patient experienced regression of bothersome symptoms and improved erections. A 2-year follow-up revealed sustained symptom relief. LiSWT presents a non-invasive means of inducing mechanical stress and microtrauma in targeted tissues, fostering neovascularization and potentially enhancing blood supply. The integration of LiSWT with PDE5-I and physical therapy suggests a potential avenue for effective HFS management. Nevertheless, further systematic research is essential to validate the therapy’s benefits and assess, if any, potential drawbacks.

## Introduction

Hard Flaccid Syndrome (HFS) can be a disease management challenge due to its complex nature and the lack of a standardized treatment regimen [1]. The current approach to managing HFS involves a multifaceted strategy integrating medical, physical, and psychological interventions to overcome the various symptoms associated with the condition [2, 3]. While pharmaceutical solutions are limited, available medications can relieve pain and improve erectile function [2, 4]. In addition, physical therapy, especially for the pelvic floor, has shown potential in relieving symptoms [1]. This is supported by some case reports where patients have experienced notable improvement, especially in cases involving prolonged penile pain [2].

Recognizing the gaps in the literature and the lack of large-scale clinical trials is crucial. Reliance on anecdotal evidence and case reports in HFS highlights the need for more comprehensive research and standardized reporting [2]. Doing so will help determine the effectiveness of existing interventions and lay the foundation for innovative treatment modalities. A recent survey underscores the importance of developing multimodal and personalized treatment strategies for HFS patients, given the prevailing lack of familiarity among professionals, the necessity for ongoing education and research to enhance diagnostic accuracy, and the imperative to improve patient care through effective treatment strategies [5].

This case report strives to enhance the existing knowledge, offer valuable insights, and foster a deeper comprehension of HFS. Its ultimate goal is to contribute to developing more effective and targeted interventions for this perplexing condition.

## Patient information

A 36-year-old male patient with no previous significant medical history presented to our tertiary men’s health clinic with complaints of rigidity when the penis was not erect, low libido, intermittent chronic pain around the perineum and penis, along with an inability to achieve sufficient erection and rigidity for penetration despite sexual desire on 09/09/2021. He did not previously receive any professional health care for this complaint. He attempted skin stretching exercises but noted no benefit and instead developed significant pain. Since then, the patient has had sensory issues in the penis as well as some hardness even when the penis is soft. The patient had no known medical, family, or psychosocial disease history. Also, a history of traumatic masturbation or sexual intercourse was not found.

## Clinical findings

Physical examination revealed a circumcised penis, and the patient reported tearing of foreskin and glans irritation with masturbation and sex. There was no history of any drug use. Testosterone, prolactin, and thyroid function values were in the normal reference range. Duplex findings suggested some mild corporal fibrosis but no other findings. On physical examination, there was no palpable plaque.

## Diagnostic assessment

Based on previous literature, diagnostic tests for HFS, such as blood profile, penile Doppler, and imaging methods, usually yield normal results. Diagnosis largely depends on the patient’s medical history. Anamnesis plays the most important role in making a diagnosis [2].

When making a differential diagnosis for HFS, it is essential to exclude conditions such as pelvic floor dysfunction, Peyronie’s disease, psychogenic erectile dysfunction, chronic prostatitis/chronic pelvic pain syndrome, neurological and vascular disorders, as well as potential hormonal imbalances, and past penile trauma or infections. Accurate diagnosis requires careful consideration of these conditions through detailed history taking, physical examination, and specific diagnostic tests, including hormone profile and imaging studies.

The lack of studies on HFS makes urologists inexperienced in its prognosis and management [5].

## Therapeutic intervention

Treatment was discussed and planned with the patient. Tadalafil 5 mg(Cialis®; Eli Lilly and Company, Indianapolis, Ind) was administered once a day and not to exceed one tablet per day, and LiSWT(Urogold 100 (Woodstock, GA)) was administered. LiSWT settings were: energy flux density: 0.13 mJ/mm^2^, Frequency: 3 Hz, Total number of shocks delivered: 3600, with 600 shocks delivered to the right, left, dorsal, and ventral aspects of the shaft, and the right and left crura, respectively. A total of 6 sessions of LiSWT were performed. Additionally, The patient maintained a consistent treatment schedule, attending physical therapy 1-2 times per week over a span of 10–12 weeks.

## Follow-up and outcomes

The treatment was completely tolerated without any side effects during or after the procedure. We found a significant regression in patient complaints at the end of the combination of PDE5-I, LiSWT, and physical therapy. Pelvic pain regressed significantly with physical therapy, especially at the end of LiSWT. The complaint of mild rigidity of the penis in the flaccid state completely regressed, and the erection quality improved. The patient started to achieve a sexually satisfied erection during intercourse. After two years of follow-up, the patient remained symptom-free.

## Discussion

HFS is a multifaceted urological condition that remains elusive in its etiology and clinical management [1]. A complex interaction of biological, psychological, and social factors is hypothesized, with stress often playing a pivotal role [3]. The resultant psychosexual implications of HFS can be devastating, leading to physical discomfort and significant psychological distress, including anxiety, depression, and altered self-perception [1, 4, 6].

Historically, management strategies for HFS have leaned towards pain management, stress reduction, and physical therapies [1]. However, given the need for consistent efficacy and the absence of a standardized therapeutic approach, there is a pressing need for innovative treatment modalities [2]. While shock wave therapy has shown promise in various chronic pain conditions, its application to HFS is still nascent but potentially promising [7, 8].

**Fig. 1:**
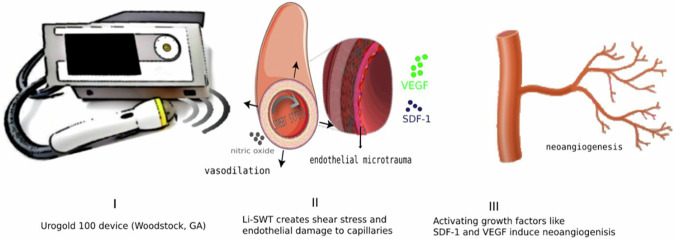
Shows the plausible mechanism of action of LiSWT.

LiSWT provides a non-invasive method to induce mechanical stress and microtrauma in targeted tissues [9]. This, in turn, can lead to a series of biological reactions, notably neovascularization by angiogenic factors [7]. The cascading effect of angiogenic factors released due to the therapy might improve blood supply and alleviate some of the symptoms of HFS [10–12] (Fig. 1).

In the presented case, a patient with a clear manifestation of HFS showed remarkable improvement after undergoing a multimodal regimen of daily tadalafil 5 mg, 6 sessions of LiSWT, and LiSWT. The decision to combine these treatments is underpinned by the belief that the pathophysiology of HFS might overlap with that of ED [1, 12]. Thus, strategies effective against ED might benefit HFS patients. Breakdown of cyclic nucleotides: cAMP and cGMP is accomplished by the enzymes PDE5-Is [13]. PDE5-I increases nitric oxide (NO) present after sexual stimulation [14]. After NO is released, inhibition of PDE5 leads to the accumulation of cGMP levels, playing a role in the relaxation of smooth muscles and the influx of blood for penile erection [14]. Concomitant treatment with PDE5-I can help improve ED, which is often present in men with HFS. Given the complex symptom cluster seen with HFS, single-modality treatment may not be successful. Multi-modal therapy with LiSWT, PDE5-I, pelvic floor therapy, and/or behavioral health interventions are likely to be the most effective treatment strategy [13].

In a recent article, a patient who presented to the clinic with complaints related to HFS was found to have an annular tear with L5-S1 disc herniation [15]. This suggested that it may be related to pathologic activation of the pelvic/pudendal hypogastric reflex and strengthened the hypothesis that it produces symptoms according to the affected anatomical area [15]. HFS occurring after trauma (sexual intercourse, masturbation) while the penis is erect indicates end organ pathology for stimulating sympathetic activity excessively. We think that we benefited from our treatment by downregulating sympathetic triggers. However, according to this etiological theory, LiSWT may be useless in diseases that may trigger this excessive sympathetic activity at another level - at the level of the pudendal nerve or cauda equina - such as blunt perineal trauma, sacral radiculopathy.

While this case provides a promising trimodal therapy, it is essential to note that the results represent only one individual’s response. A larger sample size and randomized clinical trials will be essential to validate the therapy’s benefits and potential drawbacks.

In conclusion, while HFS remains a challenging condition in men’s health, applying physical therapy and LiSWT combined with daily 5 mg PDE5-I offers a reassuring, effective treatment.
